# Sample Size Requirements to Test Subgroup-Specific Treatment Effects in Cluster-Randomized Trials

**DOI:** 10.1007/s11121-023-01590-6

**Published:** 2023-10-10

**Authors:** Xueqi Wang, Keith S. Goldfeld, Monica Taljaard, Fan Li

**Affiliations:** 1 Department of Biostatistics, Yale School of Public Health, New Haven, CT USA; 2 Section of Geriatrics, Department of Internal Medicine, Yale School of Medicine, New Haven, CT USA; 3 Division of Biostatistics, Department of Population Health, NYU Grossman School of Medicine, New York, NY USA; 4 Clinical Epidemiology Program, Ottawa Hospital Research Institute, Ottawa, Canada; 5 School of Epidemiology and Public Health, University of Ottawa, Ottawa, Canada; 6 Center for Methods in Implementation and Prevention Science, Yale School of Public Health, Suite 200, Room 229, 135 College Street, New Haven, CT 06510 USA

**Keywords:** Gerontology, Health equity, Heterogeneity of treatment effect, Intersection–union test, Omnibus test, Power analysis

## Abstract

Cluster-randomized trials (CRTs) often allocate intact clusters of participants to treatment or control conditions and are increasingly used to evaluate healthcare delivery interventions. While previous studies have developed sample size methods for testing confirmatory hypotheses of treatment effect heterogeneity in CRTs (i.e., targeting the difference between subgroup-specific treatment effects), sample size methods for testing the subgroup-specific treatment effects themselves have not received adequate attention—despite a rising interest in health equity considerations in CRTs. In this article, we develop formal methods for sample size and power analyses for testing subgroup-specific treatment effects in parallel-arm CRTs with a continuous outcome and a binary subgroup variable. We point out that the variances of the subgroup-specific treatment effect estimators and their covariance are given by weighted averages of the variance of the overall average treatment effect estimator and the variance of the heterogeneous treatment effect estimator. This analytical insight facilitates an explicit characterization of the requirements for both the omnibus test and the intersection–union test to achieve the desired level of power. Generalizations to allow for subgroup-specific variance structures are also discussed. We report on a simulation study to validate the proposed sample size methods and demonstrate that the empirical power corresponds well with the predicted power for both tests. The design and setting of the Umea Dementia and Exercise (UMDEX) CRT in older adults are used to illustrate our sample size methods.

**Supplementary Information:**

The online version contains supplementary material available at 10.1007/s11121-023-01590-6.

## Introduction

Pragmatic cluster-randomized trials (CRTs) are commonly conducted in healthcare delivery systems and adopt cluster randomization due to logistical, administrative, or political considerations (Turner et al., 2017a, 2017b). While the overall average treatment effect has been the primary focus in many CRTs, there is an emerging interest in understanding whether the intervention is effective in pre-specified participant subgroups, such as those defined by baseline demographics or clinical characteristics (Bowden et al., 2021; Cox & Kelcey, 2022; Dong et al., 2018, 2021a, b; Gabler et al., 2009; Kravitz et al., 2004; Li & Konstantopoulos, 2023; Spybrook et al., 2016). Participant subgroups can respond to the intervention differently for various reasons, such as differential access to healthcare and differences in clinical characteristics. With an increasing number of CRTs conducted under routine healthcare conditions with the inclusion of broader eligible populations, there is also a greater need to assess how participant-level or cluster-level factors moderate the intervention effect, facilitating the development of interventions to reduce known health disparities and improve health equity. Subgroup analyses based on health equity variables are not uncommon in pragmatic trials. For example, Nicholls et al. (2023) reviewed 62 pragmatics trials of people with dementia published from 2014 to 2019 and identified 10 studies reporting subgroup analyses across health equity variables; the majority of these studies employed an interaction test. In addition, Starks et al. (2019) conducted a systematic review of CRTs published between 1/1/2010 and 3/29/2016 that focused on cardiovascular disease, chronic lower respiratory disease, and cancer. They reported that 16 out of 64 CRTs examined heterogeneity of treatment effects among demographic participant subgroups but noted a lack of guidance on subgroup analyses for CRTs. Given this context, statistical methods that address sample size and power considerations with a focus on subgroup-specific treatment effects (also referred to as stratum-specific effects by epidemiologists or sometimes simple main effects by social scientists) in pragmatic CRTs are vitally important but remain relatively underdeveloped.

There have been several recent efforts to develop explicit sample size methods for testing confirmatory hypotheses about treatment effect heterogeneity in CRTs. For example, assuming a linear mixed analysis of the covariance model, Yang et al. (2020) proposed an analytical sample size expression for the treatment-by-covariate interaction test and pointed out that results depend on both the intracluster correlation coefficient (ICC) of the outcome and that of the covariate (or sometimes referred to as the effect modifier). The ICC measures the degree of similarity between outcomes measured within the same cluster and plays an important role in planning CRTs (Eldridge et al., 2009). Tong et al. (2022) relaxed the equal cluster size assumption and investigated the impact of variable cluster sizes on power for an interaction test in CRTs. They found that the coefficient of variation of the cluster size (defined as the standard deviation of cluster size divided by the mean cluster size) has minimal impact on the variance of the interaction effect estimator, as long as the effect modifier is measured at the participant level. Li et al. (2022) generalized these sample size procedures for testing heterogeneity of treatment effect to accommodate three-level CRTs with randomization carried out at either the cluster or subcluster level.

While these prior efforts have primarily focused on sample size requirements for testing differences between subgroup-specific treatment effects, sample size methods for testing the subgroup-specific treatment effects themselves have not received adequate attention. In principle, the test of the subgroup-specific treatment effect addresses the question of whether the intervention is effective in one or more subpopulations, as defined, for example, by sex, race, baseline comorbidities, or other health equity variables. In addition, the power analysis for detecting an intervention effect in any subgroup or all subgroups may not be the same as applying a standard power evaluation for an overall effect but with a smaller sample size, because the target hypotheses can be different and because a common practice for data analysis proceeds with an analysis of covariance model including a treatment-by-subgroup interaction that provides a unifying analysis framework for assessment of both overall average treatment effect and subgroup-specific treatment effects (Yang et al., 2020). To fill this important methodological gap, we propose formal sample size procedures for testing subgroup-specific treatment effects in CRTs based on the linear mixed analysis of the covariance model. We focus on a continuous outcome and a binary subgroup variable (measured either at the participant level or cluster level). We outline explicit expressions for power and its key determinants when the focus is on subgroup-specific treatment effects. Finally, we carry out a simulation study to validate our analytical expressions under different target null and alternative hypotheses relevant to subgroup analyses in CRTs.

Our proposed sample size methods are illustrated in the context of the Umea Dementia and Exercise (UMDEX) study (Toots et al., 2016), a CRT evaluating a high-intensity functional exercise program versus a seated control activity to reduce decline in independence in activities of daily living (ADLs) among older people with dementia in residential care facilities. To reduce the risk of contamination, naturally occurring clusters consisting of residents with cognitive impairment who were inhabitants of the same wing, unit, or floor were randomized to receive the intervention or the control (both delivered at the cluster level). Specifically, the study involved 36 clusters of 3 to 8 participants each and considered a continuous primary outcome. To detect potential differences in exercise effects among subpopulations defined by dementia type, prespecified subgroup analyses by dementia type were performed. Dementia type was dichotomized as Alzheimer’s versus non-Alzheimer’s dementia (including vascular, mixed Alzheimer’s and vascular, frontotemporal, Lewy body, and Parkinson’s dementia), as the majority of previous trials only included individuals with Alzheimer’s disease (Toots et al., 2016). We will formally quantify the sample size required to achieve sufficient power for subgroup analyses. Although dementia type is not a traditional health-equity effect modifier, it defines important subgroups in gerontological research. Additionally, we would like to emphasize that the proposed methods can be applied to any binary effect modifier including typical health equity variables such as race/ethnicity and socioeconomic status.

## Methods

### Linear Mixed Analysis of the Covariance Model

We consider a CRT with $n$ clusters, where ${n}_{1}$ clusters are randomized to the intervention condition, and the remaining $n-{n}_{1}$ clusters to the control condition. The randomization proportion is defined as $\pi ={n}_{1}/n$. We write ${Y}_{ij}$ as the quantitative outcome of participant $j$ in cluster $i$ and denote the total number of participants in cluster $i$ as ${m}_{i} (i=1,\dots ,n)$. Furthermore, ${Z}_{i}\in \{\mathrm{0,1}\}$ denotes the treatment status for cluster $i$. Suppose ${S}_{ij}$ is a binary subgroup variable taking values in $\{\mathrm{0,1}\}$. For example, ${S}_{ij}=1$ indicates a resident with Alzheimer’s disease, and ${S}_{ij}=0$ indicates a resident with non-Alzheimer’s dementia in the UMDEX study (or ${S}_{ij}$ could be referred to as a binary demographic variable such as sex in other contexts). A common analytical model for examining the subgroup-specific treatment effect in CRTs is the analysis of the covariance model:

1 $$\begin{array}{c}{Y}_{ij}={\beta }_{1}+{\beta }_{2}{Z}_{i}+{\beta }_{3}{S}_{ij}+{\beta }_{4}{Z}_{i}{S}_{ij}+{b}_{i}+{\epsilon }_{ij},\end{array}$$

where ${b}_{i}\sim N\left(0,{\sigma }_{b}^{2}\right)$ and ${\epsilon }_{ij}\sim N\left(0,{\sigma }_{\epsilon }^{2}\right)$ are the random cluster-level intercept and error, respectively. In addition, ${\beta }_{1}$ is the intercept, ${\beta }_{2}=E\left[{Y}_{ij}|{Z}_{i}=1,{S}_{ij}=0\right]-E\left[{Y}_{ij}|{Z}_{i}=0,{S}_{ij}=0\right]={\Delta }_{0}$, which is the treatment effect among the subgroup defined by the collection of indices ${\mathbb{S}}_{0}=\left\{\left(i,j\right);{S}_{ij}=0\right\}$ (in the UMDEX study, ${\mathbb{S}}_{0}$ refers to the subgroup of participants with Alzheimer’s disease), ${\beta }_{3}$ is the main effect of the subgroup variable, and ${\beta }_{4}$ is the treatment-by-subgroup interaction. The model also implies the treatment effect among the subgroup ${\mathbb{S}}_{1}=\left\{\left(i,j\right), {S}_{ij}=1\right\}$ as ${\beta }_{2}+{\beta }_{4}= E\left[{Y}_{ij}|{Z}_{i}=1,{S}_{ij}=1\right]-E\left[{Y}_{ij}|{Z}_{i}=0,{S}_{ij}=1\right]={\Delta }_{1}$. Specifically, previous research focused on testing for ${\beta }_{4}$, whereas this paper focuses on testing for $\left({\Delta }_{0},{\Delta }_{1}\right)$. In Model (1), the total variance of the outcome is defined as ${\sigma }_{y|s,z}^{2}={\sigma }_{b}^{2}+{\sigma }_{\epsilon }^{2}$ and the ICC of the outcome is given by the ratio of the between-cluster variance and the total variance, or ${\rho }_{y|s,z}=\frac{{\sigma }_{b}^{2}}{{\sigma }_{b}^{2}+{\sigma }_{\epsilon }^{2}}$ (Eldridge et al, 2009). Estimating the subgroup-specific treatment effects $\left({\Delta }_{0},{\Delta }_{1}\right)$ requires estimating the regression parameters, which typically proceeds via maximum likelihood techniques. In the design stage, sample size calculations often assume that the variance components (and hence the ICC) are known and require an explicit characterization of the variance expressions. Specifically, if we represent the collection of design points for each participant as ${X}_{ij}={\left(1,{Z}_{i},{S}_{ij},{Z}_{i}{S}_{ij}\right)}^{T}$ and the design matrix for each cluster as ${X}_{i}={\left({X}_{i1},\dots {X}_{i{m}_{i}}\right)}^{T}$, then the best unbiased linear estimator for regression coefficients $\beta ={\left({\beta }_{1},{\beta }_{2},{\beta }_{3},{\beta }_{4}\right)}^{T}$ is given by $\widehat{\beta }={\left({\sum }_{i=1}^{n}{X}_{i}^{T}{V}_{i}^{-1}{X}_{i}\right)}^{-1}\left({X}_{i}^{T}{V}_{i}^{-1}{Y}_{i}\right)$, where ${V}_{i}={\sigma }_{y|s,z}^{2}\left\{\left(1-{\rho }_{y|s,z}\right){I}_{{m}_{i}}+{\rho }_{y|s,z}{J}_{{m}_{i}}\right\}$ is the compound symmetric variance matrix (${I}_{{m}_{i}}$ is the ${m}_{i}\times {m}_{i}$ identity matrix and ${J}_{{m}_{i}}$ is the ${m}_{i}\times {m}_{i}$ matrix of ones), and ${Y}_{i}={\left({Y}_{i1},\dots ,{Y}_{i{m}_{i}}\right)}^{T}$ is the collection of all outcomes in cluster $i$. The estimators for the subgroup-specific treatment effects are then given by ${\widehat{\Delta }}_{0}={\widehat{\beta }}_{2}$ and ${\widehat{\Delta }}_{1}={\widehat{\beta }}_{2}+{\widehat{\beta }}_{4}$, whose variance expressions are of interest for study design calculations. To obtain these variances, it is useful to study the variance–covariance matrix for $\widehat{\beta }$, given by ${\Sigma }_{n}={\left({\sum }_{i=1}^{n}{X}_{i}^{T}{V}_{i}^{-1}{X}_{i}\right)}^{-1}$. For simplicity, we make the conventional assumption of equal cluster sizes such that ${m}_{i}=m$ for all $i$.

### Variance for Subgroup-Specific Treatment Effect Estimators

We first review key existing results to set the stage for introducing our new results. Assuming Model (1), Yang et al. (2020) developed an explicit expression for $Var\left({\widehat{\beta }}_{4}\right)$, which was referred to as the variance of the heterogeneous treatment effect estimator. The variance for this treatment-by-subgroup interaction effect estimator takes the following explicit form.

2 $$\begin{array}{c}{\sigma }_{HTE}^{2}=Var\left({\widehat{\beta }}_{4}\right)=\frac{{\sigma }_{y}^{2}\left(1-{\rho }_{y|s,z}\right)\left\{1+\left(m-1\right){\rho }_{y|s,z}\right\}}{\pi \left(1-\pi \right){p}_{1}{p}_{0}nm\left\{1+\left(m-2\right){\rho }_{y|s,z}-\left(m-1\right){\rho }_{s}{\rho }_{y|s,z}\right\}},\end{array}$$

where ${p}_{1}=P[{S}_{ij}=1]$ is the marginal probability of the subgroup population ${\mathbb{S}}_{1}$, and ${p}_{0}=1-{p}_{1}$ is the marginal probability of the subgroup population ${\mathbb{S}}_{0}$. Three key observations follow. First, ${\sigma }_{HTE}^{2}$ depends on both the ICC of the outcome adjusting for the subgroup variable (${\rho }_{y|s,z}$), as well as the ICC of the subgroup variable itself (${\rho }_{s}$). In CRTs, both the outcome of interest and baseline covariates can have non-zero ICCs (Raudenbush, 1997), and when interest lies in detecting heterogeneity of treatment effects, these two ICC parameters play an equally important role in determining study power. Second, while ${\sigma }_{HTE}^{2}$ is monotonically increasing in ${\rho }_{s}$, it has a parabolic relationship with ${\rho }_{y|s,z}$; therefore, a larger value of the outcome ICC does not always inflate ${\sigma }_{HTE}^{2}$ when holding all other parameters constant (Yang et al., 2020). Thirdly, as a special case of Eq. (2), when the subgroup variable is measured at the cluster level (cluster subgroups are formed such that ${S}_{ij}={S}_{i}$ for all $j$), we have ${\rho }_{s}=1$, and expression (2) reduces to a much simpler expression ${\widetilde{\sigma }}_{HTE}^{2}=Var({\widehat{\beta }}_{4})=\frac{{\sigma }_{y}^{2}\{1+\left(m-1\right){\rho }_{y|s,z}\}}{\pi (1-\pi ){p}_{1}{p}_{0}nm}$*.* More generally, Tong et al. (2022) have shown that under Model (1) with the subgroup variable measured either at the participant level or cluster level, the variance for the overall average treatment effect estimator (overall average treatment effect parameter defined as ${{p}_{1}{\Delta }_{1}+{p}_{0}{\Delta }_{0}=\beta }_{2}+{p}_{1}{\beta }_{4}$) is

3 $$\begin{array}{c}{\sigma }_{ATE}^{2}=Var\left({\widehat{\beta }}_{2}+{p}_{1}{\widehat{\beta }}_{4}\right)=\frac{{\sigma }_{y}^{2}\left\{1+\left(m-1\right){\rho }_{y|s,z}\right\}}{\pi \left(1-\pi \right)nm}={p}_{1}{p}_{0}{\widetilde{\sigma }}_{HTE}^{2},\end{array}$$

which also has the identical form to the variance of an average treatment effect estimator without the subgroup variable (Murray, 1998), with the caveat that the outcome ICC is now defined adjusting for the subgroup indicator. While these variance expressions have been characterized in prior literature, we provide new insights pertaining to the variances of the subgroup-specific treatment effect estimators in Result 1. Brief derivation details are found in Web Appendix A.

**Result 1**. *Under Model (1) with the subgroup variable measured either at the participant level or cluster level, the variances of the subgroup-specific treatment effect estimators and their covariance are given by weighted averages of the variance of the overall average treatment effect estimator and the variance of the heterogeneous treatment effect estimator; that is,*

$$Var\left({\widehat{\Delta }}_{0}\right)={\sigma }_{ATE}^{2}+{p}_{1}^{2}{\sigma }_{HTE}^{2}, Var\left({\widehat{\Delta }}_{1}\right)={\sigma }_{ATE}^{2}+{p}_{0}^{2}{\sigma }_{HTE}^{2},$$

$$Cov\left({\widehat{\Delta }}_{0},{\widehat{\Delta }}_{1}\right)={\sigma }_{ATE}^{2}-{p}_{1}{p}_{0}{\sigma }_{HTE}^{2},$$

where ${\sigma }_{ATE}^{2}$ and ${\sigma }_{HTE}^{2}$ are defined above in Eqs. (2) and (3) respectively.

Several observations follow from Result 1. Firstly, the variance of each subgroup-specific treatment effect estimator does not depend on the true effect size and is only a function of ${\sigma }_{ATE}^{2}$, ${\sigma }_{HTE}^{2}$ as well as the marginal prevalence of the subgroup indicator, ${p}_{1}$. As expected, a larger subgroup (e.g., ${\mathbb{S}}_{1}$ increases in size when ${p}_{1}$ moves closer to 1) corresponds to a smaller variance of the associated subgroup-specific treatment effect estimator. When ${p}_{1}=0.5$, the two subgroup sizes are balanced in expectation such that $Var\left({\widehat{\Delta }}_{0}\right)=Var\left({\widehat{\Delta }}_{1}\right)={\sigma }_{ATE}^{2}+{\frac{1}{4}\sigma }_{HTE}^{2}$. Secondly, the covariance between the subgroup-specific treatment effect estimators is the difference between the variance of the overall average effect estimator and that of the heterogeneous effect estimator scaled by ${p}_{1}{p}_{0}$. In the extreme case where the subgroup variable is defined at the cluster level (in which case the covariate ICC ${\rho }_{s}=1$, then $Cov\left({\widehat{\Delta }}_{0},{\widehat{\Delta }}_{1}\right)={\sigma }_{ATE}^{2}-{{p}_{1}{p}_{0}\widetilde{\sigma }}_{HTE}^{2}=0$ and the two subgroup effect estimators are uncorrelated (just like conducting two separate studies), regardless of the subgroup proportions. In this case, $Var\left({\widehat{\Delta }}_{0}\right)={\sigma }_{ATE}^{2}/{p}_{0}$ and $Var\left({\widehat{\Delta }}_{1}\right)={\sigma }_{ATE}^{2}/{p}_{1}$ and the variance of the subgroup treatment effect estimator is inversely proportional to the size of the subgroup; in addition, $Var\left({\widehat{\Delta }}_{0}\right)+Var\left({\widehat{\Delta }}_{1}\right)={\widetilde{\sigma }}_{HTE}^{2}$. Finally, if we set ${{\rho }_{s}=\rho }_{y|s,z}=0$, the result is applicable for subgroup analyses in individually randomized trials where data are often assumed to be independent.

### Sample Size Estimation Based on Omnibus Test

The explicit characterization of the covariance matrix for $\left({\widehat{\Delta }}_{0},{\widehat{\Delta }}_{1}\right)$ provides an analytically tractable approach for quantifying the power for testing the subgroup-specific treatment effects. We first consider the null hypothesis that the intervention has no effect in both subgroups, corresponding to testing ${H}_{0}:{\Delta }_{0}={\Delta }_{1}=0$ versus ${H}_{1}:{\Delta }_{0}\ne 0$ and/or ${\Delta }_{1}\ne 0$. In this case, an investigator may declare the treatment a success if an effect is observed in at least one subgroup. A possible test statistic for ${H}_{0}$ is the $F$-statistic, given by the quadratic form ${F}^{*}=\left({\widehat{\Delta }}_{0},{\widehat{\Delta }}_{1}\right){\widehat{\Omega }}_{\Delta }^{-1}{\left({\widehat{\Delta }}_{0},{\widehat{\Delta }}_{1}\right)}^{T}/2$, where ${\widehat{\Omega }}_{\Delta }$ is the estimated covariance matrix of the subgroup-specific treatment effect estimators (${\widehat\Delta }_{0},{\widehat\Delta }_{1}$) with elements defined in Result 1 (an explicit expression is before Eq. (4)). Under ${H}_{0}$, ${F}^{*}$ approximately follows a central $F$-distribution with the numerator and denominator degrees of freedom $\left(2,n-2\right)$, where $n-2$ was chosen as the between-within degrees of freedom (# of clusters − # of cluster-level covariates) to reflect a penalty due to at least two cluster-level parameters in Model (1); an alternative choice of degrees of freedom, such as $n-4$, can be made with a cluster-level subgroup variable. We consider the $F$-test rather than the ${\chi }^{2}$-test because the former usually has a more robust small-sample performance (Roy et al., 2007; Tian et al., 2022). Under the alternative, ${F}^{*}$ approximately follows a non-central $F$-distribution with noncentrality parameter $\lambda =\left({\Delta }_{0},{\Delta }_{1}\right){\Omega }_{\Delta }^{-1}{\left({\Delta }_{0},{\Delta }_{1}\right)}^{T}$, where we have obtained from Result 1 that

$${\Omega }_{\Delta }=Var\left[{\left({\widehat{\Delta }}_{0},{\widehat{\Delta }}_{1}\right)}^{T}\right]={\sigma }_{ATE}^{2}{J}_{2}+{\sigma }_{HTE}^{2}\left[\begin{array}{cc}{p}_{1}^{2}& -{p}_{1}{p}_{0}\\ -{p}_{1}{p}_{0}& {p}_{0}^{2}\end{array}\right]$$

Therefore, for a nominal type I error rate $\alpha$, the power under a given effect size $\left({\Delta }_{0},{\Delta }_{1}\right)$ is

4 $$\begin{array}{c}power=1-\gamma ={\int }_{{F}_{1-\alpha }\left(2,n-2\right)}^{\infty }f\left(x;\lambda ,2,n-2\right)dx,\end{array}$$

where ${F}_{1-\alpha }(2,n-2)$ is the critical value of the central $F(2,n-2)$ distribution, and $f(x;\lambda ,2,n-2)$ refers to the probability density function of the noncentral $F(\lambda ,2,n-2)$ distribution. Finally, to determine the required sample size, one could fix the type I error rate ($\alpha$), randomization proportion ($\pi$), subgroup proportions (${p}_{1}, {p}_{0}$), outcome variance (${\sigma }_{y|s,z}^{2}$), outcome ICC (${\rho }_{y|s,z}$), subgroup variable ICC (${\rho }_{s}$), cluster size ($m$), and effect sizes (${\Delta }_{0},{\Delta }_{1}$) and specify a series of integers $n$. Then the required sample size can be obtained as the smallest integer that provides the pre-specified power ($1-\gamma$) using Eq. (4). The role of $n$ and $m$ can be switched in this procedure to solve for the required cluster size given the number of clusters.

### Sample Size Estimation Based on Intersection–union Test

Alternatively, a more stringent testing framework can be considered such that the null hypothesis would only be rejected when there is a treatment effect in both subgroups. That is, an investigator would declare the intervention a success only if a treatment effect is observed in both subgroups. In this case, one may be interested in testing ${H}_{0}: {\Delta }_{0}=0$ and/or ${\Delta }_{1}=0$ versus ${H}_{1}:{\Delta }_{0}\ne 0$ and ${\Delta }_{1}\ne 0$ and employ the intersection–union test based on the linear mixed analysis of the covariance model. Of note, the null space is composite as it includes the following three cases: treatment has no effect on both subgroups, treatment has no effect on subgroup ${\mathbb{S}}_{0}$, and treatment has no effect on subgroup ${\mathbb{S}}_{1}$. For testing this composite null, we consider the bivariate Wald test statistic, $\zeta ={\left({\zeta }_{0},{\zeta }_{1}\right)}^{T}$, where ${\zeta }_{0}=\sqrt{n}{\widehat{\Delta }}_{0}/\widehat{SE}\left({\widehat{\Delta }}_{0}\right)$ and ${\zeta }_{1}=\sqrt{n}{\widehat{\Delta }}_{1}/\widehat{SE}\left({\widehat{\Delta }}_{1}\right)$ represent the standard error–adjusted treatment effect estimators. Therefore, one can show that $\zeta$ follows a multivariate normal distribution with mean $\eta ={\left(\sqrt{n}{{\left\{Var\left({\widehat{\Delta }}_{0}\right)\right\}}^{-1/2}\Delta }_{0},\sqrt{n}{{\left\{Var\left({\widehat{\Delta }}_{1}\right)\right\}}^{-1/2}\Delta }_{1}\right)}^{T}$ and correlation matrix $\Phi$, whose diagonal elements are given by 1 and off-diagonal elements by ${\left\{Var\left({\widehat{\Delta }}_{0}\right)\right\}}^{-1/2}Cov\left({\widehat{\Delta }}_{0},{\widehat{\Delta }}_{1}\right){\left\{Var\left({\widehat{\Delta }}_{1}\right)\right\}}^{-1/2}$ (Tian et al., 2022). Given the total number of clusters $n$ and cluster size $m$, the power function to simultaneously detect the treatment effect in both subgroups is given by

5 $$\begin{array}{c}power=1-\lambda =P\left\{{\zeta }_{0}>{c}_{0}, {\zeta }_{1}>{c}_{1}|{H}_{1}\right\}={\int }_{{c}_{o}}^{\infty }{\int }_{{c}_{1}}^{\infty }g\left(a,b\right)dadb,\end{array}$$

where $\left\{{c}_{0},{c}_{1}\right\}$ are two subgroup-specific critical values for rejecting the null, and $g(a,b)$ is the density function of the Wald test statistics under the alternative. While a typical choice of $g$ is the multivariate normal distribution, we follow Yang et al. (2022) and consider a bivariate $t$-distribution with location vector $\eta$, shape matrix $\Phi$, and degrees of freedom $n-2$ as this has been shown to have better control of type I error rates in small samples (by partially accounting for the variability in estimating the covariance parameters). The specification of critical values can lead to intersection–union tests with different operating characteristics (Kordzakhia et al., 2010), and we adopt a simple approach such that ${c}_{0}={c}_{1}={t}_{\alpha }\left(n-2\right)$, which is the $\left(1-\alpha \right)$ quantile of the univariate $t$-distribution. That is, we reject ${H}_{0}$ when ${\zeta }_{0}>{t}_{\alpha }\left(n-2\right)$ and ${\zeta }_{1}>{t}_{\alpha }\left(n-2\right)$. This specification of critical values is at most conservative such that the type I error rate is controlled to be strictly below $\alpha$ within the composite null space (Li et al., 2020). Of note, the performance of this approach can critically depend on the number of clusters. For example, when the number of clusters is small, the estimated degrees of freedom $n-2$ may be very small, and therefore, the test may be conservative (Davis‐Plourde et al., 2023). Finally, for sample size determination, one can use the power Eq. (5) and solve for $n$ or $m$ given pre-specified values of all other design parameters using the procedure described for the omnibus test.

### The Role of ICC Parameters

**Fig. 1:**
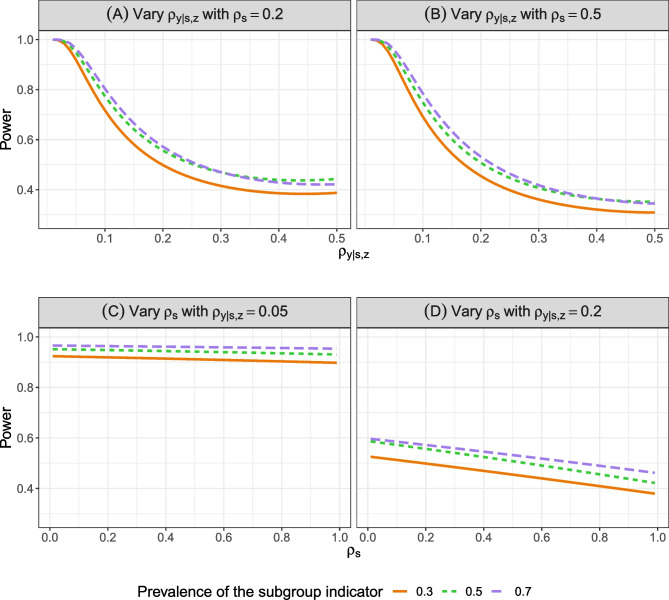
Power of the *omnibus test* with $n=30, m=100, {\sigma }_{y}^{2}=1, {\Delta }_{0}=0.3, {\Delta }_{1}=0.4$ at ${p}_{1}\in \{0.3, 0.5, 0.7\}$ as a function of (A) ${\rho }_{y|s,z}$ when fixing ${\rho }_{s}=0.2$; (B) ${\rho }_{y|s,z}$ when fixing ${\rho }_{s}=0.5$; (C) ${\rho }_{s}$ when fixing ${\rho }_{y|s,z}=0.05$; (D) ${\rho }_{s}$ when fixing ${\rho }_{y|s,z}=0.2$

**Fig. 2:**
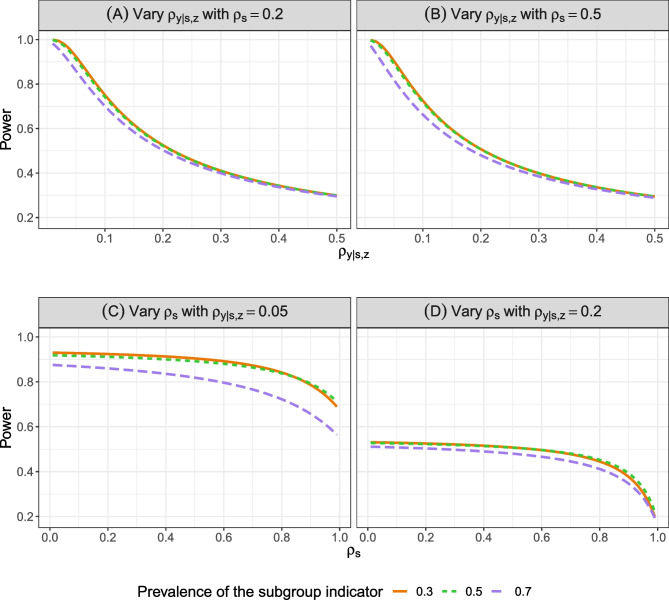
Power of the *intersection–union test* with $n=30, m=100, {{\upsigma }_{\mathrm{y}}^{2}=1,\Delta }_{0}=0.3, {\Delta }_{1}=0.4$ at ${p}_{1}\in \{0.3, 0.5, 0.7\}$ as a function of (A) ${\rho }_{y|s,z}$ when fixing ${\rho }_{s}=0.2$; (B) ${\rho }_{y|s,z}$ when fixing ${\rho }_{s}=0.5$; (C) ${\rho }_{s}$ when fixing ${\rho }_{y|s,z}=0.05$; (D) ${\rho }_{s}$ when fixing ${\rho }_{y|s,z}=0.2$

To provide some intuition on how the ICC parameters affect study power, we numerically explore the relationship between power and the two relevant ICC parameters (${\rho }_{y|s,z}$ and ${\rho }_{s}$) for the omnibus test and intersection–union test in Figs. 1 and 2. We consider a CRT with equal allocation to both arms with $\pi =1/2$ and assume $n=30$ clusters, cluster size $m=100$, total variance of the outcome ${\sigma }_{y|s,z}^{2}=1$, the treatment effect among the subgroup ${\mathbb{S}}_{0}$ is ${\Delta }_{0}=0.3$, and the treatment effect among the subgroup ${\mathbb{S}}_{1}$ is ${\Delta }_{1}=0.4$ and vary the prevalence of the subgroup indicator by choosing ${p}_{1}\in \{0.3, 0.5, 0.7\}$. In Fig. 1, we observe that the power of the omnibus test monotonically decreases in ${\rho }_{s}$ but has a parabolic relationship with ${\rho }_{y|s,z}$. In general, power is not too sensitive to ${\rho }_{s}$, especially when ${\rho }_{y|s,z}$ is small. But power often increases as the prevalence of the subgroup with a larger treatment effect increases. In Fig. 2, we observe that the power of the intersection–union test monotonically decreases in both ${\rho }_{s}$ and ${\rho }_{y|s,z}$, is more sensitive to changes in ${\rho }_{y|s,z}$ than in ${\rho }_{s}$, and appears to be more sensitive to changes in ${\rho }_{s}$ than the omnibus text, particularly for larger values of ${\rho }_{s}$. In practice, we recommend exploring the sensitivity of sample size and power under varying a priori estimates for the two ICC parameters, as our numerical results illustrate that power may change according to different ICC assumptions.

## Simulation Study

### Simulation Design

We follow the ADEMP framework proposed by Morris et al. (2019), which breaks down the simulation study into five key elements: aims, data-generating mechanisms, estimands, methods, and performance measures.

#### Aims

This simulation study aims to assess the performance of our sample size formulas with equal randomization ($\pi =1/2$) and equal subgroup proportions (${p}_{1}={p}_{0}=1/2$), for both the omnibus test and the intersection–union test. The primary objectives are to verify that the empirical type I error rate is controlled at or under the nominal level and empirical power is close to that predicted by the formula.

#### Data-Generating Mechanisms

From the proposed sample size formulas, the total number of clusters depends on the following parameters: nominal type I error rate ($\alpha$), power ($1-\lambda$), total variance of the outcome (${\sigma }_{y|s,z}^{2}$), ICC of the outcome (${\rho }_{y|s,z}$), ICC of the subgroup variable (${\rho }_{s}$), cluster size ($m$), and effect sizes. Throughout, we fixed the total variance ${\sigma }_{y|s,z}^{2}$ at 1, nominal type I error rate $\alpha$ at 5%, desired power $1-\lambda$ at 80%, ${\beta }_{2}=0.2$ and ${\beta }_{4}=0.1$ for the omnibus test, and ${\beta }_{2}=0.3$ and ${\beta }_{4}=0.1$ for the intersection–union test and varied the remaining parameters. That is, the true subgroup-specific treatment effects were ${\Delta }_{0}=0.2$ and ${\Delta }_{1}=0.3$ for the omnibus test and ${\Delta }_{0}=0.3$ and ${\Delta }_{1}=0.4$ for the intersection–union test. Different effect sizes were chosen for different tests to ensure a realistic range of the predicted number of clusters. We considered three levels of cluster size $m\in \{20, 50, 100\}$, three levels of ICC for the outcome conditional on the subgroup variable ${\rho }_{y|s,z}\in \{0.02, 0.05, 0.1\}$, and three levels of ICC for the subgroup variable ${\rho }_{s}\in \{0.1, 0.25, 0.5\}$; ${\rho }_{s}$ was chosen to follow previous simulations for the interaction tests (Tong et al., 2022; Yang et al., 2020) and to better illustrate its potential impact on power. In summary, we considered $3\times 3\times 3=27$ parameter combinations for each test. In each scenario, the total number of clusters $n$ was determined as the smallest number that ensured the predicted power was at least 80%. To assess the empirical type I error rate, both ${\beta }_{2}$ and ${\beta }_{4}$ were fixed at 0 for the omnibus test, while only ${\beta }_{2}$ was fixed at 0 for the intersection–union test. For each sample size obtained from the respective formula, we then simulated the binary subgroup variable ${S}_{ij}$ from the beta-binomial model with the cluster-specific probability of the subgroup population ${\mathbb{S}}_{1}$ as ${p}_{i}\sim Beta({q}_{1}, {q}_{2})$ and ${S}_{ij}\sim Bernoulli({p}_{i})$, where ${q}_{1}$ and ${q}_{2}$ were determined by the marginal probability of the subgroup population ${\mathbb{S}}_{1}$ as ${p}_{1}={q}_{1}/({q}_{1}+{q}_{2})$ and the ICC of the subgroup variable as ${\rho }_{s}={\left(1+{q}_{1}+{q}_{2}\right)}^{-1}$. For each scenario, we also simulated the outcome ${Y}_{ij}$ from Model (1), by fixing ${\beta }_{1}=0$ and ${\beta }_{3}=0.15$ (in theory the power is not affected by these two parameters).

#### Estimands

Given our focus on testing, the estimand aspect of the ADEMP framework could be interpreted as the empirical power and empirical type I error rate of each test, estimated by the formula predictions.

#### Methods

In each scenario, 2000 data replications were generated and analyzed for the evaluation of the empirical type I error rate under the null and empirical power under the alternative hypothesis. As the nominal type I error rate was 5%, according to the margin of error from a binomial model with 2000 replications, we considered an empirical type I error rate from 4.0 to 6.0% as close to the nominal. Similarly, as the predicted power was at least 80% for each scenario, we considered an empirical power differing at most 2.0% from the predicted power as acceptable. In addition, for each scenario, we also provide a comparison with a back-of-the-envelope approach. This approach estimates the required number of clusters ${n}_{c}$ by first using our formula ignoring any intracluster correlations with ${\rho }_{y|s,z}={\rho }_{s}=0$ (i.e., assuming individual randomization) and then multiplying the required sample size by the conventional design effect for CRTs: $1+\left(m-1\right){\rho }_{y|s,z}$. We also calculate the actual predicted power using our formula based on ${n}_{c}$ to compare the performance of the back-of-the-envelope approach to our method, as well as the relative saving in the required number of clusters ($=\frac{{n}_{c}-n}{{n}_{c}}\times 100\%$).

#### Performance Measures

To assess the performance of the sample size formulas for each test, we compute both the empirical type I error rate and empirical power in each simulation scenario. The empirical type I error rate is calculated as the percentage of times a null hypothesis is rejected when the null is actually true; the empirical power is calculated as the percentage of times a null hypothesis is rejected when the null is actually false.

### Simulation Results

All statistical analyses were conducted with R, version 4.2.2, and the convergence rate was 100% for each scenario. Table 1 summarizes the estimated required number of clusters ($n$) using the proposed formula, empirical type I error, empirical power, and predicted power, for the *omnibus test*. For all scenarios, the type I error rates were all within the acceptable range, and the empirical power corresponded well with the predicted power. In the last three columns of Table 1, we present the results for the back-of-the-envelope approach. We observe that for the omnibus test, simply inflating the sample size with the conventional design effect always leads to a larger sample size than the proposed method, and the relative savings in the required number of clusters ranges from $8.3$ to $54.5\%$ across the scenarios considered.

**Table 1:** Simulation scenarios^a^, estimated required number of clusters $n$ based on the proposed formula, empirical type I error rates (emp. size), empirical power (emp. power), and predicted power (pred. power) for the *omnibus test*. The treatment effect among the subgroup ${\mathbb{S}}_{0}$ is ${\Delta }_{0}=0.2$, and the treatment effect among the subgroup ${\mathbb{S}}_{1}$ is ${\Delta }_{1}=0.3$. In the last two columns, we estimate the required number of clusters ${n}_{c}$ using the proposed formula with ${\rho }_{y|s,z}={\rho }_{s}=0$ and the design effect with the true value of ${\rho }_{y|s,z}$ and then obtain the actual predicted power (actual power) using our formula based on ${n}_{c}$ as well as the true values of ${\rho }_{y|s,z}$ and ${\rho }_{s}$

Design parameters				Performance characteristics^b^			Comparator		
$m$	${\rho }_{y|s,z}$	${\rho }_{s}$	$n$	Emp. size	Emp. power	Pred. power	${n}_{c}$	Actual power	Rel. saving (%)
20	0.02	0.10	44	0.046	0.795	0.806	48	0.843	8.3
		0.25	44	0.045	0.805	0.805	48	0.842	8.3
		0.50	44	0.045	0.797	0.803	48	0.841	8.3
	0.05	0.10	60	0.046	0.818	0.808	68	0.859	11.8
		0.25	60	0.055	0.800	0.806	68	0.857	11.8
		0.50	60	0.060	0.791	0.802	68	0.854	11.8
	0.10	0.10	84	0.044	0.818	0.804	100	0.874	16.0
		0.25	86	0.048	0.816	0.810	100	0.870	14.0
		0.50	86	0.043	0.812	0.802	100	0.863	14.0
50	0.02	0.10	26	0.043	0.797	0.804	32	0.890	18.8
		0.25	26	0.044	0.793	0.801	32	0.889	18.8
		0.50	28	0.043	0.829	0.832	32	0.885	12.5
	0.05	0.10	42	0.046	0.813	0.815	56	0.920	25.0
		0.25	42	0.046	0.816	0.809	56	0.916	25.0
		0.50	44	0.045	0.805	0.820	56	0.910	21.4
	0.10	0.10	62	0.049	0.792	0.801	96	0.947	35.4
		0.25	64	0.047	0.808	0.803	96	0.941	33.3
		0.50	68	0.048	0.803	0.811	96	0.931	29.2
100	0.02	0.10	20	0.042	0.818	0.808	30	0.953	33.3
		0.25	20	0.047	0.802	0.804	30	0.951	33.3
		0.50	22	0.042	0.844	0.842	30	0.947	26.7
	0.05	0.10	34	0.043	0.834	0.814	60	0.977	43.3
		0.25	34	0.044	0.819	0.803	60	0.974	43.3
		0.50	36	0.047	0.820	0.811	60	0.968	40.0
	0.10	0.10	50	0.041	0.815	0.801	110	0.992	54.5
		0.25	54	0.052	0.809	0.816	110	0.989	50.9
		0.50	58	0.045	0.810	0.812	110	0.982	47.3

^a^All scenarios assume a CRT with equal randomization ($\pi =0.5$) and equal subgroup proportions (${p}_{1}={p}_{0}=0.5$) and a quantitative outcome having variance ${\sigma }_{y}^{2}=1$

^b^The type I error rates were all within the acceptable range (from $4.0\%$ to $6.0\%$), and the empirical power corresponded well with the predicted power (differing at most $2.0\%$ from the preficted power)

Table 2 summarizes the estimated required number of clusters ($n$) using the proposed formula, empirical type I error, empirical power, and predicted power, for the *intersection–union test*. With a small cluster size ($m=20, 50$), the intersection–union test provided conservative type I error rates ($<4.0\%$); for the larger cluster size ($m=100$), the type I error rates grew closer to nominal. The empirical power corresponded well with the predicted power across almost all scenarios. Finally, we also observe that for the intersection–union test, inflating the sample size under individual randomization via the simple design effect always results in a larger number of clusters than the proposed method and may therefore lead to unnecessary use of resources. Specifically, the relative savings in the required number of clusters ranges from $17.4$ to $59.1\%$ across the scenarios considered. Therefore, our method is especially attractive when the available numbers of clusters or resources are limited.

**Table 2:** Simulation scenarios^a^, estimated required number of clusters $n$ based on the proposed formula, empirical type I error rates (emp. size), empirical power (emp. power), and predicted power (pred. power) for the *intersection–union test*. The treatment effect among the subgroup ${\mathbb{S}}_{0}$ is ${\Delta }_{0}=0.3$, and the treatment effect among the subgroup ${\mathbb{S}}_{1}$ is ${\Delta }_{1}=0.4$. In the last two columns, we estimate the required number of clusters ${n}_{c}$ using the proposed formula with ${\rho }_{y|s,z}={\rho }_{s}=0$ and the design effect with the true value of ${\rho }_{y|s,z}$ and then obtain the actual predicted power (actual power) using our formula based on ${n}_{c}$ as well as the true values of ${\rho }_{y|s,z}$ and ${\rho }_{s}$

Design parameters				Performance characteristics^b^			Comparator		
m	${\rho }_{(y|s,z)}$	${\rho }_{s}$	*n*	Emp. size	Emp. power	Pred. power	${n}_{c}$	Actual power	Rel. saving (%)
20	0.02	0.10	38	0.020*	0.802	0.811	46	0.883	17.4
		0.25	38	0.016*	0.805	0.803	46	0.876	17.4
		0.50	40	0.022*	0.794	0.810	46	0.864	13.0
	0.05	0.10	46	0.024*	0.815	0.809	64	0.919	28.1
		0.25	48	0.028*	0.814	0.814	64	0.911	25.0
		0.50	50	0.024*	0.800	0.805	64	0.894	21.9
	0.10	0.10	58	0.030*	0.799	0.802	94	0.946	38.3
		0.25	60	0.035*	0.809	0.802	94	0.940	36.2
		0.50	66	0.024*	0.816	0.810	94	0.924	29.8
50	0.02	0.10	20	0.026*	0.821	0.818	28	0.929	28.6
		0.25	20	0.027*	0.795	0.805	28	0.922	28.6
		0.50	22	0.028*	0.806	0.821	28	0.906	21.4
	0.05	0.10	28	0.035*	0.824	0.812	50	0.967	44.0
		0.25	30	0.032*	0.805**	0.826	50	0.962	40.0
		0.50	32	0.035*	0.820	0.822	50	0.951	36.0
	0.10	0.10	42	0.040	0.814	0.816	84	0.978	50.0
		0.25	42	0.037*	0.813	0.806	84	0.976	50.0
		0.50	46	0.038*	0.812	0.815	84	0.969	45.2
100	0.02	0.10	14	0.033*	0.822	0.825	24	0.970	41.7
		0.25	14	0.030*	0.792**	0.813	24	0.966	41.7
		0.50	16	0.032*	0.822	0.840	24	0.956	33.3
	0.05	0.10	22	0.042	0.809	0.815	48	0.987	54.2
		0.25	22	0.037*	0.789	0.805	48	0.985	54.2
		0.50	24	0.036*	0.819	0.814	48	0.980	50.0
	0.10	0.10	36	0.059	0.823	0.816	88	0.992	59.1
		0.25	36	0.057	0.828	0.810	88	0.991	59.1
		0.50	38	0.043	0.810	0.813	88	0.989	56.8

^a^All scenarios assume a CRT with equal randomization ($\pi =0.5$) and equal subgroup proportions (${p}_{1}={p}_{0}=0.5$) and a quantitative outcome having variance ${\sigma }_{y}^{2}=1$

^b^Starred text indicates the type I error rates were smaller than $4.0\%$, which were conservative but the tests were still valid; double starred text indicates the empirical power was more than $2.0\%$ smaller than the predicted power

## Illustrative Data Example

We illustrate our sample size methods by calculating the required number of clusters (i.e., groups of participants) in the context of the UMDEX study, which was introduced in the “Introduction” section. Recall that an important aim of the study was to investigate intervention effects in different subpopulations defined by dementia type: Alzheimer’s disease versus non-Alzheimer’s dementia. Clusters were randomized to either the exercise or control activities in a 1:1 ratio. The intervention exercise program consisted of five exercise sessions lasting approximately 45 min, each held per 2-week period for 4 months (40 sessions in total). The primary outcome of ADL independence was measured at the patient level at 4 months using the FIM, a 13-item instrument with items rated on a scale from total assistance (1) to complete independence (7) and a total score ranging from 13 to 91. We treat the FIM as a continuous outcome with larger values indicating more independence in ADLs.

**Fig. 3:**
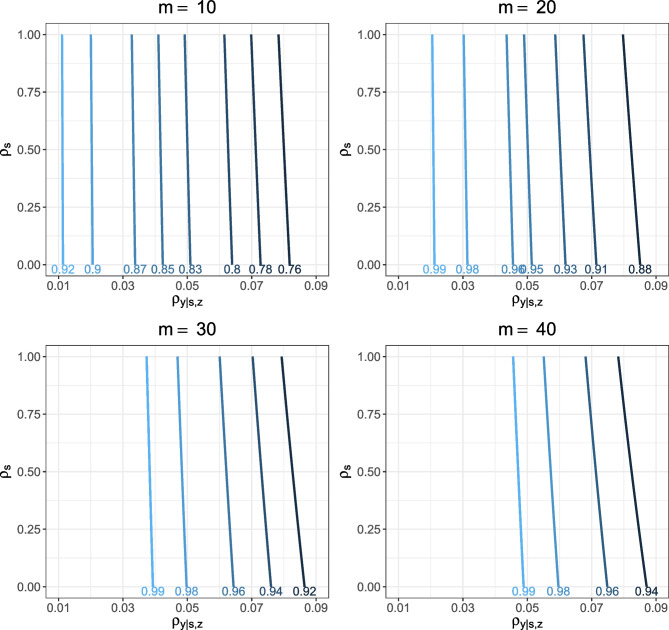
Predicted power contours for the *omnibus test* as a function of ${\rho }_{s}$ and ${\rho }_{y|s,z}$ at $m=\{10, 20, 30, 40\}$, with $n=18$, ${\Delta }_{0}=0.7\times {\sigma }_{y}$, ${\Delta }_{1}=0.5\times {\sigma }_{y}$ for the UMDEX study

First, suppose the investigators were interested in the omnibus test, demonstrating a treatment effect in at least one of the two subgroups. They need to determine the required number of clusters to achieve at least 80% power at the 5% nominal test size. The target effect size for the subgroup with non-Alzheimer’s dementia was a standardized difference of ${\Delta }_{0}/{\sigma }_{y|s,z}=0.7$ and for the subgroup with Alzheimer’s disease was ${\Delta }_{1}/{\sigma }_{y|s,z}=0.5.$ Furthermore, the assumed ICC of the subgroup variable (dementia type) was ${\rho }_{s}=0.2$, and of the outcome FIM adjusted for the subgroup indicator was ${\rho }_{y|s,z}=0.04$. The anticipated prevalence of patients with Alzheimer’s disease was ${p}_{1}=36\%$, and the anticipated number of patients per cluster was assumed to be $m=10$. With these assumptions and inverting power Eq. (4), the required number of clusters can be calculated as 18 with a predicted power of 85.5%. Figure 3 shows a sensitivity analysis of power as a function of ${\rho }_{s}$ and ${\rho }_{y|s,z}$ and for $m=\{10, 20, 30, 40\}$, with $n=18$. When ${\rho }_{y|s,z}\le 0.09$, the predicted power decreases as ${\rho }_{s}$ or ${\rho }_{y|s,z}$ increases. Overall, even with 10 patients per cluster, the power of the study remains above 0.8 as long as ${\rho }_{y|s,z}\le 0.06$. However, using the back-of-the-envelope approach, the required number of clusters would be 20 (i.e., 2 more clusters compared to the proposed method) with an actual power of 89.8%.

**Fig. 4:**
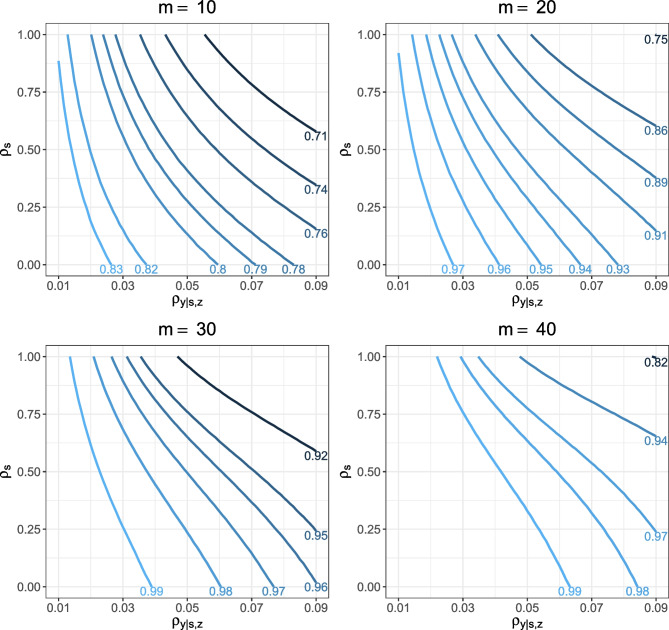
Predicted power contours for the *intersection–union test* as a function of ${\rho }_{s}$ and ${\rho }_{y|s,z}$ at $m=\{10, 20, 30, 40\}$, with $n=34$, ${\Delta }_{0}=0.7\times {\sigma }_{y}$, ${\Delta }_{1}=0.5\times {\sigma }_{y}$ for the UMDEX study

Second, suppose the investigators were interested in the intersection–union test, demonstrating treatment effects in both subgroups. Again, they need to determine the required number of clusters to achieve at least 80% power at the 5% nominal test size. Keeping all of the other assumptions as in the omnibus test and using Eq. (5), the required number of clusters would be 34 with a power of 80.6%. With the same assumptions, more clusters are required for the intersection–union test because of a more stringent requirement of treatment effects in both subgroups based on the omnibus test. Figure 4 shows a sensitivity analysis of power as a function of ${\rho }_{s}$ and ${\rho }_{y|s,z}$ at $m=\{10, 20, 30, 40\}$, assuming $n=34$. When ${\rho }_{y|s,z}\le 0.09$, the predicted power decreases as ${\rho }_{s}$ or ${\rho }_{y|s,z}$ increases, and the power appears to be more sensitive to ${\rho }_{s}$ compared to the omnibus test. Overall, even with 10 patients per cluster, the power of the study remains above 0.8 as long as ${\rho }_{y|s,z}\le 0.04$ and ${\rho }_{s}\le 0.25$. However, using the back-of-the-envelope approach, the required number of clusters would be 42 (i.e., 8 more clusters compared to the proposed method) with an actual power of 87.7%.

In this example, we additionally consider the sample size required for the interaction test (Yang et al., 2020). Suppose the investigators were interested in the interaction test for heterogeneity of treatment effects, demonstrating a difference in treatment effects between the two subgroups. Keeping all of the other assumptions as in the omnibus test and the intersection–union test and using the method in Yang et al. (2020), the required number of clusters would be 284 with a power of 80.2%. Note that the interaction test requires a much larger sample size because the effect size for the between-subgroup difference is much smaller than the effect size for each subgroup. This comparison demonstrates that sample size requirements for subgroup-specific treatment effects and those for testing treatment effect heterogeneity do not have a clear nesting relationship, and the choice between them ultimately depends on the scientific study objective. Finally, we calculated the required sample size for testing the overall average treatment effect (${H}_{0}:{\beta }_{2}+{p}_{1}{\beta }_{4}=0$ versus ${H}_{1}:{\beta }_{2}+{p}_{1}{\beta }_{4}\ne 0$). Keeping all assumptions as above, using a $t$-test with the variance (3) suggested that the required number of clusters would be 12 with a power of 85.9%; thus, a smaller sample size is sufficient for testing the overall average treatment effect.

## Discussion

It is increasingly important for investigators to explicitly formulate health equity objectives about testing subgroup-specific treatment effects and then design the trial accordingly, i.e., with adequate power to address the health equity objectives. Accordingly, there is an emerging need to study sample size requirements for such objectives, especially for cluster-randomized designs. Recently, the National Institute on Aging (NIA) IMbedded Pragmatic Alzheimer’s disease (AD) and AD-Related Dementias (AD/ADRD) Clinical Trials (IMPACT) Collaboratory considered the need to “clearly state health-equity-relevant aims & hypotheses” and “be explicit in sample size justification with regard to the health equity objective” in their health equity best practices guidance document (NIA IMPACT Collaboratory, 2022). It is therefore critically important to integrate health equity considerations in the design stage of a pragmatic trial (Nicholls et al., 2023), for which we contribute analytical power and sample size formulas. We consider a simple yet common case with a binary subgroup variable and clarify the ingredients that determine the variance of the subgroup-specific treatment effect under a linear mixed analysis of the covariance model. On some occasions, we recognize that many CRTs include the analysis of the overall treatment effect as the primary analysis and the subgroup analysis as secondary. In those cases, our methods can help provide a context to interpret the subgroup results and address questions of how many more clusters are needed if the study aims to generate evidence on subgroup treatment effects. Alternatively, if the sample size is driven solely by the overall average treatment effect, it is still helpful to know what power is available to detect plausible subgroup treatment effects, even if it is not $80\%$. Moreover, we consider both the omnibus test and the intersection–union test. The choice of tests depends on the study context and scientific question, and our work allows investigators to focus on either test, as well as compare the sample size implications of the two tests. In addition, for the omnibus test, power generally decreases in the ICC of the subgroup variable and has a parabolic relationship with the ICC of the outcome conditional on the subgroup variable; for the intersection–union test, power monotonically decreases in both ICC parameters. This information can help investigators specify ICC parameters that are likely to provide conservative sample size estimates if accurate information on design parameters is unavailable at the design stage. Finally, even though our data example does not include a multilevel intervention which has two or more levels of intervention at the same time or in close temporal proximity (Agurs-Collins et al., 2019), our approach remains applicable to a multilevel intervention as long as the study considers cluster-level randomization.

Importantly, this paper focuses on testing subgroup-specific treatment effects and has a distinct focus from the previous research on the heterogeneity of treatment effects (i.e., an interaction test). In our perspective, these two analyses provide complementary evidence by addressing different aspects of how intervention affects subpopulations in CRTs. The choice between testing subgroup-specific treatment effects and testing heterogeneity of treatment effects ultimately depends on the study objective, that is, whether the study aims to test the treatment effect in each subgroup or the difference in treatment effects between the subgroups. The required sample size to detect the subgroup-specific treatment effects is expected to be different than that for testing the treatment difference, even under the same effect size specifications, as demonstrated in the illustrative example. The reason is that our methods depend on the size of effects specified for each subgroup, while the interaction test depends only on the difference between two subgroups. Furthermore, there exist practical situations with an unbalanced distribution of an important subgroup variable (e.g., gender identity) whereby the trial could only include very few people in one group but many more in another. This might lead to challenges in powering the interaction test (since ${p}_{1}{p}_{0}$ is close to zero), but one may still have a chance to identify treatment effect signals in at least one subgroup with sufficient power. Given the importance of addressing sex-gender considerations in trial designs, our methodology allows investigators to ensure that there is adequate power in at least the larger of the two subgroups even when the study may not be adequately powered for detecting heterogeneity of treatment effects.

Although Model (1) is a commonly used analytical model, it assumes that the correlation among participant outcomes within the same cluster is the same between the two subgroups. Ignoring the difference in correlations among members of the same cluster in the two subgroups, when it exists, may lead to an inflated type I error rate. Extending Model (1), we can include a random coefficient for ${S}_{ij}$ to allow the outcome correlation among participants from the same cluster to differ between subgroups. Specifically, one can consider the model:

6 $$\begin{array}{c}{Y}_{ij}={\beta }_{1}+{\beta }_{2}{Z}_{i}+{\beta }_{3}{S}_{ij}+{\beta }_{4}{Z}_{i}{S}_{ij}+{b}_{i}+{c}_{i}{S}_{ij}+{e}_{ij}, \end{array}$$

where the parameters are similarly interpreted as those in Model (1), except for the addition of the random cluster-level slope, ${c}_{i}\sim N(0, {\sigma }_{c}^{2})$. Model (6) encodes three outcome ICCs: the ICC between different participants in subgroup ${\mathbb{S}}_{0}$, the ICC between different participants in different subgroups, and the ICC between different participants in subgroup ${\mathbb{S}}_{1}$. The closed-form formulas for $Var\left({\widehat{\Delta }}_{0}\right)$, $Var\left({\widehat{\Delta }}_{1}\right)$, and $Cov\left({\widehat{\Delta }}_{0},{\widehat{\Delta }}_{1}\right)$ under Model (6) are analytically less tractable due to the complexity of the correlation structure. Therefore, in Web Appendix B, we propose an efficient Monte-Carlo sample size procedure through simulating data and inverting the correlation matrix, as an extension to allow for different outcome ICCs in different subgroups.

Our development of sample size procedures focusing on testing subgroup-specific treatment effects for a binary subgroup variable represents an endeavor to improve standards for confirmatory subgroup analyses in CRTs but comes with several limitations that we plan to address in future work. First, there are scenarios where more than two subgroups are of interest, and our method can be generalized to accommodate multiple subgroups following the derivations in Sect. 3.2 of Yang et al. (2020). However, the final covariance matrix for the subgroup-specific treatment estimators may depend on two ICC parameters of two dummy variables, and the sample size procedure is inevitably more complicated. In addition, for a total study sample size, an increasing number of subgroups will on average lead to smaller subgroup sample sizes, which could diminish power. In future work, it would be worth exploring the implications of the number of subgroups on study power for both the omnibus and intersection–union testing frameworks. Second, we assumed equal cluster sizes to simplify derivations. Such an assumption is routinely made in CRTs at the design stage but could be violated in practice. It would be interesting to extend our sample size formulas to accommodate variable cluster sizes, perhaps along the lines of van Breukelen et al. (2007) and Tong et al. (2022). Third, our work assumed the effect of the treatment group variable is constant across clusters. An extension of our work to varying effects can be made by including an additional random slope for ${Z}_{i}$ in Model (1) or Model (6) (Tong et al., 2023). A Monte-Carlo procedure similar to that developed in Web Appendix B can be used for sample size estimation but requires assumptions on additional ICC parameters which will be explored in future work. Fourth, we only considered equal subgroup proportions in the simulation study. In addition, smaller numbers of clusters, such as 8, 10, or 12, which may occur in some CRTs, were not considered in the simulation study. Possible challenges and additional scenarios with unequal subgroup proportions and with a small number of clusters will be addressed in future work. Finally, we have assumed that the outcome is continuous, and analysis is based on linear mixed analysis of covariance. An extension of our work to binary and categorical outcomes will be pursued in future work. However, in some cases, the sample size results developed for continuous outcomes can still be used to provide an approximate calculation for binary outcomes, providing that the target effect size is on the risk difference scale. The accuracy of this approximate procedure remains to be investigated in the context of subgroup analyses.

### Supplementary Information

Below is the link to the electronic supplementary material.

Supplementary file1 (DOCX 27 KB)

## Data Availability

Source code to reproduce results in the simulation study and application are openly available on GitHub at https://github.com/XueqiWang/SubgroupATE_CRT.
