# Correction: Sample Size Requirements to Test Subgroup-Specific Treatment Effects in Cluster-Randomized Trials

**DOI:** 10.1007/s11121-023-01615-0

**Published:** 2024-01-05

**Authors:** Xueqi Wang, Keith S. Goldfeld, Monica Taljaard, Fan Li

**Affiliations:** 1 Department of Biostatistics, Yale School of Public Health, New Haven, CT USA; 2 Section of Geriatrics, Department of Internal Medicine, Yale School of Medicine, New Haven, CT USA; 3 Division of Biostatistics, Department of Population Health, NYU Grossman School of Medicine, New York, NY USA; 4 Clinical Epidemiology Program, Ottawa Hospital Research Institute, Ottawa, Canada; 5 School of Epidemiology and Public Health, University of Ottawa, Ottawa, Canada; 6 Center for Methods in Implementation and Prevention Science, Yale School of Public Health, Suite 200, Room 229, 135 College Street, New Haven, CT 06510 USA

**Correction to: Prevention Science** **https://doi.org/10.1007/s11121-023-01590-6**

The article “Sample Size Requirements to Test Subgroup-Specific Treatment Effects in Cluster-Randomized Trials”, written by Wang, X., Goldfeld, K.S., Taljaard, M., and Li, F., was originally published electronically on the publisher’s internet portal on 10 October 2023 without open access. With the author(s)’ decision to opt for Open Choice the copyright of the article changed on 01 November 2023 to © The Author(s) 2023 and the article is forthwith distributed under a Creative Commons Attribution 4.0 International License, which permits use, sharing, adaptation, distribution and reproduction in any medium or format, as long as you give appropriate credit to the original author(s) and the source, provide a link to the Creative Commons licence, and indicate if changes were made. The images or other third party material in this article are included in the article’s Creative Commons licence, unless indicated otherwise in a credit line to the material. If material is not included in the article’s Creative Commons licence and your intended use is not permitted by statutory regulation or exceeds the permitted use, you will need to obtain permission directly from the copyright holder. To view a copy of this licence, visit http://creativecommons.org/licenses/by/4.0/.

The original article has been corrected.

