# Supplementary material for: Sample Size Requirements to Test Subgroup-Specific Treatment Effects in Cluster-Randomized Trials
Source: Prev Sci. 2023 Oct 10;25(Suppl 3):356–70. doi: 10.1007/s11121-023-01590-6 (PMC11004667; doi:10.1007/s11121-023-01590-6)
Supplement: Supplementary file 1 — Supplementary file1 (DOCX 27 KB) [file 11121_2023_1590_MOESM1_ESM.docx]

**Supporting Information for “Sample size requirements to test subgroup average treatment effects in cluster randomized trials” by Wang et al.**

**Web Appendix A**

**Derivation of RESULT 1.**

Following the expressions by Yang et al. (2020) and using the block matrix inversion, we have $Var\left( \hat{\beta}_{4} \right)=\frac{\sigma_{y|s}^{2}}{\pi\left( 1-\pi\right)m\{c\left( \mu_{2}-\mu_{1}^{2} \right)+dm(\eta_{2}-\mu_{1}^{2})\}}$, where $\mu_{1}$, $\mu_{2}$, and $\eta_{2}$ are moment values of the subgroup variable as defined in Section 3.1 of Yang et al. (2020), $c=\frac{1}{1-\rho_{y|s}}$, and $d=-\frac{\rho_{y|s}}{\left( 1-\rho_{y|s} \right)\{1+(m-1)\rho_{y|s}\}}$. With a few steps of algebra, we obtain $Var\left( \hat{\beta}_{4} \right)=\sigma_{HTE}^{2}$.

Similarly, we obtain $Var\left( \hat{\beta}_{2} \right)=\sigma_{ATE}^{2}+p_{1}^{2}\sigma_{HTE}^{2}$, and $Cov\left( \hat{\beta}_{2}, \hat{\beta}_{4} \right)=-p_{1}\sigma_{HTE}^{2}$.

Therefore, we have $Var\left( \hat{\Delta}_{0} \right)=Var\left( \hat{\beta}_{2} \right)=\sigma_{ATE}^{2}+p_{1}^{2}\sigma_{HTE}^{2},$ $Var\left( \hat{\Delta}_{1} \right)={Var\left( \hat{\beta}_{2}+\hat{\beta}_{4} \right)=\sigma}_{ATE}^{2}+p_{0}^{2}\sigma_{HTE}^{2},$ and $Cov\left( \hat{\Delta}_{0},\hat{\Delta}_{1} \right)=Cov\left( \hat{\beta}_{2}, \hat{\beta}_{2}+\hat{\beta}_{4} \right)=\sigma_{ATE}^{2}-p_{1}p_{0}\sigma_{HTE}^{2}.$

**Web Appendix B**

**Extension of Model (1)**

Although Model (1) is a commonly-used analytical model, it assumes that the correlation among individual outcomes within the same cluster is the same between the two subgroups. Ignoring the difference of correlation among members of the same cluster in the two subgroups may lead to an inflated type I error rate. As an extension of Model (1), we include a random coefficient for $S_{ij}$ to allow the outcome correlation among individuals from the same cluster to differ between subgroups. Specifically, we consider the model:

$$\begin{aligned} Y_{ij}=\beta_{1}+\beta_{2}Z_{i}+\beta_{3}S_{ij}+\beta_{4}Z_{i}S_{ij}+b_{i}+c_{i}S_{ij}+e_{ij}, \#\left( 6 \right) \end{aligned}$$

where the model parameters are similarly interpreted as those in Model (1) of the main paper, except for the addition of the random cluster-level slope, $c_{i}\sim N(0, \sigma_{c}^{2})$. From this model, the total variance of the outcome for individuals in the subgroup $\mathbb{S}_{0}$ is defined as $\sigma_{y|s, 0}^{2}=\sigma_{b}^{2}+\sigma_{\epsilon}^{2}$, while the total variance of the outcome for individuals in the subgroup $\mathbb{S}_{1}$ is defined as $\sigma_{y|s, 1}^{2}=\sigma_{b}^{2}+\sigma_{c}^{2}+\sigma_{\epsilon}^{2}$. Different from Model (1) in the main paper, Model (6) encodes three distinct outcome ICCs:

(i) the ICC between different individuals in the same subgroup $\mathbb{S}_{0}$ is

$$corr\left( Y_{ij}, Y_{ij^{'}} | {Z_{ij}= Z_{ij^{'}}=0, S}_{ij}, S_{ij^{'}} \right)= \rho_{0}=\frac{\sigma_{b}^{2}}{\sigma_{b}^{2}+\sigma_{\epsilon}^{2}}, for j\neq j';$$

(ii) the ICC between different individuals in different subgroups is

$$corr\left( Y_{ij}, Y_{ij^{'}} | {Z_{ij}+ Z_{ij^{'}}=1, S}_{ij}, S_{ij^{'}} \right)= \rho_{01}=\frac{\sigma_{b}^{2}}{{{[(\sigma}_{b}^{2}+\sigma_{\epsilon}^{2})(\sigma_{b}^{2}+\sigma_{c}^{2}+\sigma_{\epsilon}^{2})]}^{1/2}}, for j\neq j';$$

(iii) the ICC between different individuals in the same subgroup $\mathbb{S}_{1}$ is

$$corr\left( Y_{ij}, Y_{ij^{'}} | {Z_{ij}= Z_{ij^{'}}=1, S}_{ij}, S_{ij^{'}} \right)= \rho_{1}=\frac{\sigma_{b}^{2}+\sigma_{c}^{2}}{\sigma_{b}^{2}+\sigma_{c}^{2}+\sigma_{\epsilon}^{2}}, for j\neq j^{'}.$$

In cluster $i (i=1, \ldots, n)$, denote the total number of individuals in the subgroup $\mathbb{S}_{0}$ as $m_{i0}$, and the total number of individuals in the subgroup $\mathbb{S}_{1}$ as $m_{i1}$, so we have $m_{i0}+m_{i1}=m_{i}$. In addition, we represent the collection of design points for each individual as $X_{ij}=\left( 1,Z_{i},S_{ij},Z_{i}S_{ij} \right)^{T}$, the design matrix for each cluster as $X_{i}=\left( X_{i1},\ldots{, X_{im_{0}}, X_{i,m_{0}+1}, \ldots, X}_{im_{i}} \right)^{T}$, and the collection of all outcomes in each cluster as $Y_{i}=\left( Y_{i1},\ldots, Y_{im_{0}}, Y_{i,m_{0}+1}, \ldots, Y_{im_{i}} \right)^{T}$. Then the best unbiased linear estimator for regression coefficients $\beta=\left( \beta_{1},\beta_{2},\beta_{3},\beta_{4} \right)^{T}$ is given by $\hat{\beta}=\left( \sum_{i=1}^{n} X_{i}^{T}V_{i}^{-1}X_{i} \right)^{-1}\left( X_{i}^{T}V_{i}^{-1}Y_{i} \right)$, where $V_{i}=A_{i}^{1/2}R_{i}A_{i}^{1/2}$, $A_{i}$ is a $m_{i}$-dimensional diagonal matrix with the first $m_{i0}$ diagonal elements being $\sigma_{y|s, 0}^{2}$ and the other diagonal elements being $\sigma_{y|s, 1}^{2}$, and $R_{i}$ is the correlation matrix expressed as

$$R_{i}=\left[ \begin{matrix} R_{i00} & R_{i01} \\ R_{i01}^{T} & R_{i11} \end{matrix} \right]=\left[ \begin{matrix} (1-\rho_{0})I_{m_{i0}}+\rho_{0}J_{m_{i0}} & \rho_{01}J_{m_{i0}\times m_{i1}} \\ \rho_{01}J_{m_{i1}\times m_{i0}} & (1-\rho_{1})I_{m_{i1}}+\rho_{1}J_{m_{i1}} \end{matrix} \right].$$

The estimators for the subgroup average treatment effects are then given by $\hat{\Delta}_{0}=\hat{\beta}_{2}$ and $\hat{\Delta}_{1}=\hat{\beta}_{2}+\hat{\beta}_{4}$, whose variance expressions are of interest for study design calculations. To obtain these variances, it is useful to study the variance-covariance matrix for $\hat{\beta}$, given by $\Sigma_{n}=\left( \sum_{i=1}^{n} X_{i}^{T}V_{i}^{-1}X_{i} \right)^{-1}$. So we need to invert the $R_{i}$ matrix

$$R_{i}^{-1}=\left[ \begin{matrix} E_{i} & F_{i} \\ F_{i}^{T} & G_{i} \end{matrix} \right].$$

According to the inverse of simple exchangeable correlation structure, we have

$$R_{i00}^{-1}=\frac{1}{1-\rho_{0}}I_{m_{i0}}-\frac{\rho_{0}}{\left( 1-\rho_{0} \right)\{1+{(m}_{i0}-1)\rho_{0}\}}J_{m_{i0}},$$

$$R_{i11}^{-1}=\frac{1}{1-\rho_{1}}I_{m_{i1}}-\frac{\rho_{1}}{\left( 1-\rho_{1} \right)\{1+{(m}_{i1}-1)\rho_{1}\}}J_{m_{i1}}.$$

By block matrix inverse, we have

$$E_{i}=\left( R_{i00}-R_{i01}R_{i11}^{-1}R_{i01}^{T} \right)^{-1}$$

$$=\left( \left( 1-\rho_{0} \right)I_{m_{i0}}+\rho_{0}J_{m_{i0}}-\frac{m_{i1}\rho_{01}^{2}}{1-\rho_{1}}J_{m_{i0}}+\frac{m_{i1}^{2}\rho_{1}\rho_{01}^{2}}{\left( 1-\rho_{1} \right)\{1+{(m}_{i1}-1)\rho_{1}\}} \right)^{-1}$$

$$=\left( \left( 1-\rho_{0} \right)I_{m_{i0}}+\frac{\rho_{0}{\{1+{(m}_{i1}-1)\rho_{1}\}-m_{i1}\rho}_{01}^{2}}{1+{(m}_{i1}-1)\rho_{1}}J_{m_{i0}} \right)^{-1}$$

$$=\frac{1}{1-\rho_{0}}I_{m_{i0}}-\frac{c_{i0}}{\left( 1-\rho_{0} \right)\left( 1-\rho_{0}+m_{i0}c_{i0} \right)}J_{m_{i0}},$$

where $c_{i0}=\frac{\rho_{0}{\{1+{(m}_{i1}-1)\rho_{1}\}-m_{i1}\rho}_{01}^{2}}{1+{(m}_{i1}-1)\rho_{1}}$. Similarly,

$$G_{i}=\left( R_{i11}-R_{i01}^{T}R_{i00}^{-1}R_{i01} \right)^{-1}$$

$$=\left( \left( 1-\rho_{1} \right)I_{m_{i1}}+\rho_{1}J_{m_{i1}}-\frac{m_{i0}\rho_{01}^{2}}{1-\rho_{0}}J_{m_{i1}}+\frac{m_{i0}^{2}{\rho_{0}\rho}_{01}^{2}}{\left( 1-\rho_{0} \right)\{1+{(m}_{i0}-1)\rho_{0}\}} \right)^{-1}$$

$$=\left( \left( 1-\rho_{1} \right)I_{m_{i1}}+\frac{\rho_{1}{\{1+{(m}_{i0}-1)\rho_{0}\}-m_{i0}\rho}_{01}^{2}}{1+{(m}_{i0}-1)\rho_{0}}J_{m_{i1}} \right)^{-1}$$

$$=\frac{1}{1-\rho_{1}}I_{m_{i1}}-\frac{c_{i1}}{\left( 1-\rho_{1} \right)\left( 1-\rho_{1}+m_{i1}c_{i1} \right)}J_{m_{i1}},$$

where $c_{i1}=\frac{\rho_{1}{\{1+{(m}_{i0}-1)\rho_{0}\}-m_{i0}\rho}_{01}^{2}}{1+{(m}_{i0}-1)\rho_{0}}$. In addition,

$$F_{i}=-E_{i}R_{i01}R_{i11}^{-1}$$

$$=-\frac{\rho_{01}}{\left\{ 1+{(m}_{i0}-1 \right)\rho_{0}\}\{1+{(m}_{i1}-1)\rho_{1}\}-m_{i0}m_{i1}\rho_{01}^{2}}J_{m_{i0}\times m_{i1}}.$$

However, unlike Model (1) of the main paper, the closed-form formulas for $Var\left( \hat{\Delta}_{0} \right)$, $Var\left( \hat{\Delta}_{1} \right)$, and $Cov\left( \hat{\Delta}_{0},\hat{\Delta}_{1} \right)$ (or equivalently for $Var\left( \hat{\beta}_{2} \right)$, $Var\left( \hat{\beta}_{4} \right)$ and $Cov\left( \hat{\beta}_{2}, \hat{\beta}_{4} \right)$) under Model (6) are less analytically tractable due to the complexity of the correlation structure and the multiplication between $X_{i}$ and $V_{i}^{-1}$. Therefore, we propose an efficient Monte Carlo procedure (Tong et al., 2023) to estimate the sample size and power through simulating $X_{i}$’s. Specifically, the proposed efficient Monte Carlo procedure to determine an optimal sample size includes the following 6 steps.

(1) We specify the following parameters: nominal type I error rate ($\alpha$), desired power ($1-\lambda$), randomization ratio ($\pi$), total variance of the outcome for each subgroup ($\sigma_{y|s,0}^{2}, \sigma_{y|s,1}^{2}$), ICCs of the outcome ($\rho_{0}, \rho_{01}, \rho_{1}$), subgroup proportion ($p_{0}$), ICC of the subgroup variable ($\rho_{s}$), cluster sizes ($m_{i}$), and effect sizes. We also input an even number as the initial value of the number of clusters ($n$).

(2) In each iteration $b$, simulate $Z_{i}$ and $S_{ij}$ to obtain the design matrix, invert $R_{i}$ using the closed-form of $R_{i}^{-1}$, then calculate $\sum_{i=1}^{n} \left( X_{i}^{T}V_{i}^{-1}X_{i} \right)^{(b)}$ based on simulated data and $R_{i}^{-1}$.

(3) Repeat (2) for $B$ times, then estimate $\sum_{i=1}^{n} X_{i}^{T}V_{i}^{-1}X_{i}$ by the sample average, $B^{-1}\sum_{b=1}^{B} \sum_{i=1}^{n} \left( X_{i}^{T}V_{i}^{-1}X_{i} \right)^{(b)}$. Then the variance-covariance matrix for $\hat{\beta}$ is $\Sigma^{B}=\left\{ B^{-1}\sum_{b=1}^{B} \sum_{i=1}^{n} \left( X_{i}^{T}V_{i}^{-1}X_{i} \right)^{\left( b \right)} \right\}^{-1}$. Thus $Var\left( \hat{\beta}_{2} \right)$ is the (2, 2)th element of $\Sigma^{B}$, $Var\left( \hat{\beta}_{4} \right)$ is the (4, 4)th element of $\Sigma^{B}$, and $Cov\left( \hat{\beta}_{2}, \hat{\beta}_{4} \right)$ is the (2, 4)th element of $\Sigma^{B}$. We further calculate $Var\left( \hat{\Delta}_{0} \right)=Var\left( \hat{\beta}_{2} \right)$, $Var\left( \hat{\Delta}_{1} \right)=Var\left( \hat{\beta}_{2}+\hat{\beta}_{4} \right)$, and $Cov\left( \hat{\Delta}_{0},\hat{\Delta}_{1} \right)=Cov\left( \hat{\beta}_{2}, \hat{\beta}_{2}+\hat{\beta}_{4} \right)$.

(4) Evaluate the predicted power $1-\hat{\gamma}(n)$ for a given sample size $n$, using the *F*-statistic for the omnibus test or the bivariate Wald test statistic for the intersection-union test.

(5) Test if both $1-\hat{\gamma}\left( n \right)\geq1-\gamma$ and $1-\hat{\gamma}\left( n-2 \right)<1-\gamma$ are satisfied.

(6) If the answer for step (5) is yes, we output $n$ as the required number of clusters. If the answer for step (5) is no, we increase $n$ by 2 if $1-\hat{\gamma}\left( n \right)<1-\gamma$, or decrease $n$ by 2 if $1-\hat{\gamma}\left( n-2 \right)\geq1-\gamma$, then repeat steps (2)-(6).

**REFERENCES**

Tong, J., Li, F., Harhay, M. O., & Tong, G. (2023). Accounting for expected attrition in the planning of cluster randomized trials for assessing treatment effect heterogeneity. *BMC Medical Research Methodology, 23*(1), 1-14.

Yang, S., Li, F., Starks, M. A., Hernandez, A. F., Mentz, R. J., & Choudhury, K. R. (2020). Sample size requirements for detecting treatment effect heterogeneity in cluster randomized trials. *Statistics in medicine, 39*(28), 4218-4237.
